# Best Practice of Peritoneal Dialysis-Associated Gram-Negative Peritonitis in Children: Insights From the International Pediatric Peritoneal Dialysis Network Registry

**DOI:** 10.1016/j.ekir.2024.03.031

**Published:** 2024-03-28

**Authors:** Dagmara Borzych-Dużałka, Rebeca Same, Alicia Neu, Hui Kim Yap, Enrico Verrina, Sevcan A. Bakkaloglu, Francisco Cano, Hiren Patel, Maria Szczepańska, Łukasz Obrycki, Ana Paula Spizzirri, Lisa Sartz, Karel Vondrak, Anabella Rebori, Gordana Milosevski-Lomic, Eugene Yu-hin Chan, Biswanath Basu, Andrea Lazcano Pezo, Ariane Zaloszyc, Vimal Chadha, Franz Schaefer, Bradley A. Warady

**Affiliations:** 1 Department for Pediatrics, Nephrology and Hypertension, Medical University of Gdańsk, Gdańsk, Poland; 2 University Center for Pediatrics and Adolescent Medicine, Heidelberg, Germany; 3 Children’s Hospital of Philadelphia, Philadelphia, Pennsylvania, USA; 4 Johns Hopkins University School of Medicine, Baltimore, Maryland, USA; 5 Department of Pediatrics, Yong Loo Lin School of Medicine, National University of Singapore, Singapore; 6 IRCCS Instituto Giannina Gaslini, Genoa, Italy; 7 Gazi University Hospital, Ankara, Turkey; 8 Division of Pediatric Nephrology, Hospital Dr. Luis Calvo Mackenna, Facultad de Medicina, Universidad de Chile, Santiago, Chile; 9 Nationwide Children's Hospital, Columbus, Ohio, USA; 10 Department of Pediatrics, Faculty of Medical Sciences in Zabrze, Medical University of Silesia, Katowice, Poland; 11 Children’s Memorial Health Institute, Warsaw, Poland; 12 Division of Pediatric Nephrology, Hospital for Maria Ludovica, La Plata, Argentina; 13 Department of Pediatrics, Clinical Sciences Lund, Lund University, Lund, Sweden; 14 University Hospital Motol, Prague, Czech Republic; 15 SENIAD, Montevideo, Uruguay; 16 Children’s University Hospital, Belgrade, Serbia; 17 Pediatric Nephrology Centre, Hong Kong Children’s Hospital, Kowloon Bay, Hong Kong SAR; 18 NRS Medical College and Hospital, Kolkata, India; 19 Roberto del Rio Hospital, Chile; 20 Pediatric Nephrology, Pédiatrie 1, Centre Hospitalier Universitaire de Strasbourg, France; 21 Children’s Mercy Kansas City, Kansas City, Missouri, USA

**Keywords:** children, Enterobacterales, gram-negative, PD-associated peritonitis, *Pseudomonas*

## Abstract

**Introduction:**

Gram-negative peritonitis (GNP) is associated with significant morbidity in children receiving long-term peritoneal dialysis (PD) and current treatment recommendations are based on limited data.

**Methods:**

Analysis of 379 GNP episodes in 308 children (median age 6.9 years, interquartile range [IQR]: 3.0–13.6) from 45 centers in 28 countries reported to the International Pediatric Peritoneal Dialysis Network registry between 2011 and 2023.

**Results:**

Overall, 74% of episodes responded well to empiric therapy and full functional recovery (FFR) was achieved in 82% of cases. *In vitro* bacterial susceptibility to empiric antibiotics and lack of severe abdominal pain at onset were associated with a good initial response. Risk factors for failure to achieve FFR included severe abdominal pain at onset and at 60 to 72 hours from treatment initiation (odds ratio [OR]: 3.81, 95% confidence interval [CI]: 2.01–7.2 and OR: 3.94, 95% CI: 1.06–14.67, respectively), *Pseudomonas* spp. etiology (OR: 1.73, 95% CI: 1.71–4.21]) and *in vitro* bacterial resistance to empiric antibiotics (OR: 2.40, 95% CI: 1.21–4.79); the risk was lower with the use of monotherapy as definitive treatment (OR: 0.40, 95% CI: 0.21–0.77). Multivariate analysis showed no benefit of dual antibiotic therapy for treatment of *Pseudomonas* peritonitis after adjustment for age, presenting symptomatology, 60 to 72-hour treatment response, and treatment duration. Monotherapy with cefazolin in susceptible Enterobacterales peritonitis resulted in a similar FFR rate (91% vs. 93%) as treatment with ceftazidime or cefepime monotherapy.

**Conclusion:**

Detailed microbiological assessment, consisting of patient-specific and center-specific antimicrobial susceptibility data, should guide empiric treatment. Treatment “deescalation” with the use of monotherapy and narrow spectrum antibiotics according to susceptibility data is not associated with inferior outcomes and should be advocated in the context of emerging bacterial resistance.

## Graphical abstract

**Figure:**
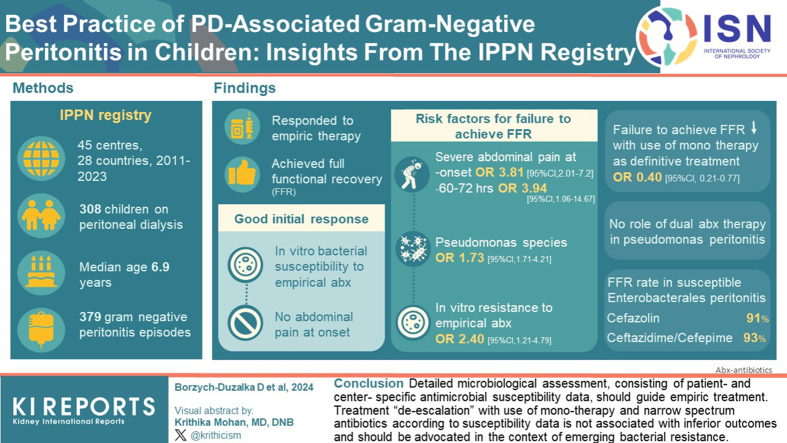

Outcomes associated with GNP in patients on long-term PD tend to be worse than for gram-positive peritonitis.^1^ Treatment of GNP can be particularly challenging due to rising antibiotic resistance and the numerous different mechanisms of resistance that can make antibiotic selection and efficacy challenging.^2^ Studies comparing different treatment strategies for specific gram-negative causes of peritonitis are lacking; thus, most recommendations are either extrapolated from general principles for the management of gram-negative infections or are based on retrospective, epidemiological data.^3^^,^^4^ As antibiotic resistance continues to expand worldwide, it is increasingly important to prioritize therapies that limit risks of resistance and adverse drug events (i.e., narrow-spectrum antibiotics and shorter treatment time), as long as these strategies are not associated with inferior outcomes.^5^ The collection of such treatment and outcome data in the “real world” setting has been carried out by the International Pediatric Peritoneal Dialysis Network since 2007. The current analysis is focused on the management of GNP. The primary end point is outcome of GNP. The intent is to subsequently incorporate what is learned from this analysis into the International Society of Peritoneal Dialysis (ISPD) pediatric guidelines for the prevention and treatment of peritonitis, which are currently being updated.^6^

## Methods

### Data Collection

The International Pediatric Peritoneal Dialysis Network Registry collects prospective information on children and adolescents treated with maintenance PD in pediatric dialysis units around the globe via a Web-based platform (www.pedpd.org). Every 6 months, anthropometric, clinical, biochemical, medication, and PD prescription data is collected, as well as information about complications and outcomes of PD. Detailed data regarding PD-related infections (peritonitis and exit site or tunnel infections) is entered for each infection episode. Peritonitis data collection includes the potential cause, clinical presentation (temperature, abdominal pain, cloudiness of effluent, as well as effluent leukocyte and its differential count), microbiological results including organisms cultured and antimicrobial susceptibilities, empiric treatment, initial (60–72-hour) treatment response, treatment modifications (definitive therapy) after receipt of culture results, and final outcome (PD continuation, FFR, and posttreatment relapses). Data entries are automatically checked for plausibility and completeness. The registry protocol was approved by institutional review boards as required at each participating center. Written parental consent and, when appropriate, assent from patients were obtained.

Study exclusion criteria included the following: (i) cumulative treatment time shorter than 7 days, (ii) PD cessation or death within 3 days of peritonitis treatment, and (iii) relapsing peritonitis.

### Definitions

In accordance with the ISPD guidelines, peritonitis is diagnosed if the patient presents with at least 2 of the following 3 criteria: (i) abdominal pain and/or cloudy dialysis effluent, (ii) dialysis effluent white cell count > 100/ml with >50% polymorphonuclear leukocytes, and (iii) positive dialysis effluent culture.^7^

Prespecified peritonitis causes include catheter perforation or leakage, accidental disconnection, poor hygiene, exit site infection, catheter insertion or other abdominal surgery, gastrostomy or percutaneous endoscopic gastrostomy tube placement, dental procedure, i.p. drug administration, and unknown. A causative relationship to a preexisting exit site infection is also assumed whenever an exit site infection preceded peritonitis by less than 30 days.

As described previously, patient temperature and severity of abdominal pain at presentation was scored and resulted in the generation of a disease severity score.^8^ The maximum temperature was characterized into 1 of 3 ranges as follows: <37.5 °C, 37.5 °C to 38.9 °C, and >38.9 °C, with corresponding scores of 0, 1, and 2, respectively. Abdominal pain was rated on a 3-point scale as absent, mild, or severe, with scores of 0, 1, and 2, respectively. A good initial clinical response was defined as a decrease in the disease severity score by ≥2 or, if less than 2 initially, a lack of cloudy effluent if initially present or a decrease in the dialysate cell count by more than 50% by 60 to 72 hours after initiation of empiric therapy.

Total treatment duration was calculated as the sum of the duration of empiric and definitive antibiotic treatment.

Final outcome was judged according to the occurrence of the following: (i) lack of FFR, (ii) PD termination, or (iii) relapse of infection. FFR was defined as PD continuation without functional impairment (i.p. adhesions and decreased ultrafiltration capacity), with or without PD catheter exchange. PD termination was defined as permanent PD discontinuation and transfer to HD or death due to a peritonitis episode. Relapse was defined as recurrence of peritonitis with the same organism that caused a prior episode within 1 month after discontinuation of antibiotic therapy.

### Statistical Analyses

Continuous variables were checked for normal distribution using the Kolmogorov-Smirnoff test and expressed as mean ± SD for normally distributed variables and median and IQR for nonnormally distributed variables. Categorical variables were expressed as frequency and percentage. Differences in proportions were assessed using χ2 test. Univariate and multivariate logistic regression analyses were applied to identify factors associated with initial treatment response, FFR, treatment failure, and relapse.

The following parameters were included in the analysis: patient age, PD duration, presence of ostomies, severity of infection at presentation and at 60 to 72 hours of treatment (temperature, abdominal pain, and cloudiness of effluent), preceding or concurrent exit site infection, bacterial strain, choice of empiric and definitive antibiotic, administration route, and treatment duration. Differences with *P* < 0.05 were considered significant. Data were analyzed using SAS, version 9.4 (SAS Institute).

## Results

Patient demographic data, dialysis duration, peritonitis cause, clinical presentation (maximal temperature and severity of abdominal pain, effluent cloudiness and cell count), culture results, antibiotics used in initial and definitive antibiotic scheme, treatment duration and final outcome were available for all 379 episodes.

### Demographics

The 379 primary GNP episodes occurred in 308 pediatric patients from 45 centers in 28 countries and were entered into the International Pediatric Peritoneal Dialysis Network database between January 1, 2011 and October 31, 2023. Of the episodes, 163 were reported from European, 83 from Asian, 58 from Latin American, 45 from Turkish and Middle East, 23 from US, and 17 from New Zealand centers. Detailed microbiological data pertaining to peritonitis etiology is presented in Table 1. Median age at presentation with peritonitis was 6.9 (IQR: 3.0–13.6) years, with 155 peritonitis episodes (41%) occurring in children aged <5 years. In 145 cases (38%), peritonitis occurred within the first 12 months of PD, in 90 (24%) within 12 to 24 months, and in 144 (35%) more than 24 months after dialysis initiation. The predominant dialysis modality was automated PD (44% nighttime intermittent PD and 33% continuous cycling PD). Of the patients, 23% received continuous ambulatory PD at peritonitis onset. Most patients had a double-cuff (90%), curled (59%) Tenckhoff catheter with a downward (43%) or laterally (43%) oriented exit site. Ninety-seven (25%) of the GNP episodes occurred in patients with ostomies, most commonly a gastrostomy (*n* = 57).

**Table 1:** Microbiology of primary gram-negative peritonitis episodes (*n* = 379)

Culture result	*n* (%)
*Pseudomonas* spp.	82 (22%)
*Acinetobacter* spp.	64 (17%)
*Klebsiella* spp.^a^	49 (14%)
*Escherichia coli*^a^	47 (12%)
*Enterobacter* spp.^a^	37 (10%)
*Serratia* spp.^a^	13 (4%)
*Moraxella* spp.	12 (3%)
*Hemophilus* spp.	9 (2%)
*Citrobacter* spp.^a^	8 (2%)
*Stenotrophomonas* spp.	8 (2%)
*Neisseria* spp.	6 (1%)
Other gram-negative	44 (11%)
Total	379 (100%)

a Enterobacterales.

### Clinical Presentation, Peritonitis Cause, and Empiric Therapy

**Figure 1:**
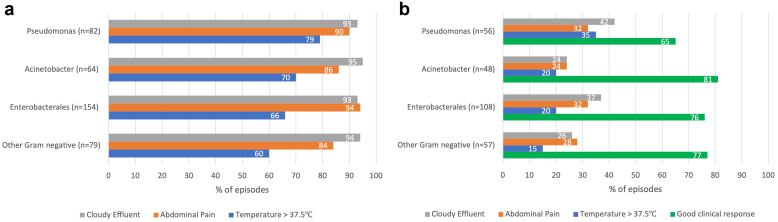
(a) Symptomatology at onset in all 379 episodes. (b). Early treatment response at 60 to 72 hours posttreatment initiation in 269 gram-negative peritonitis episodes.

**Figure 2:**
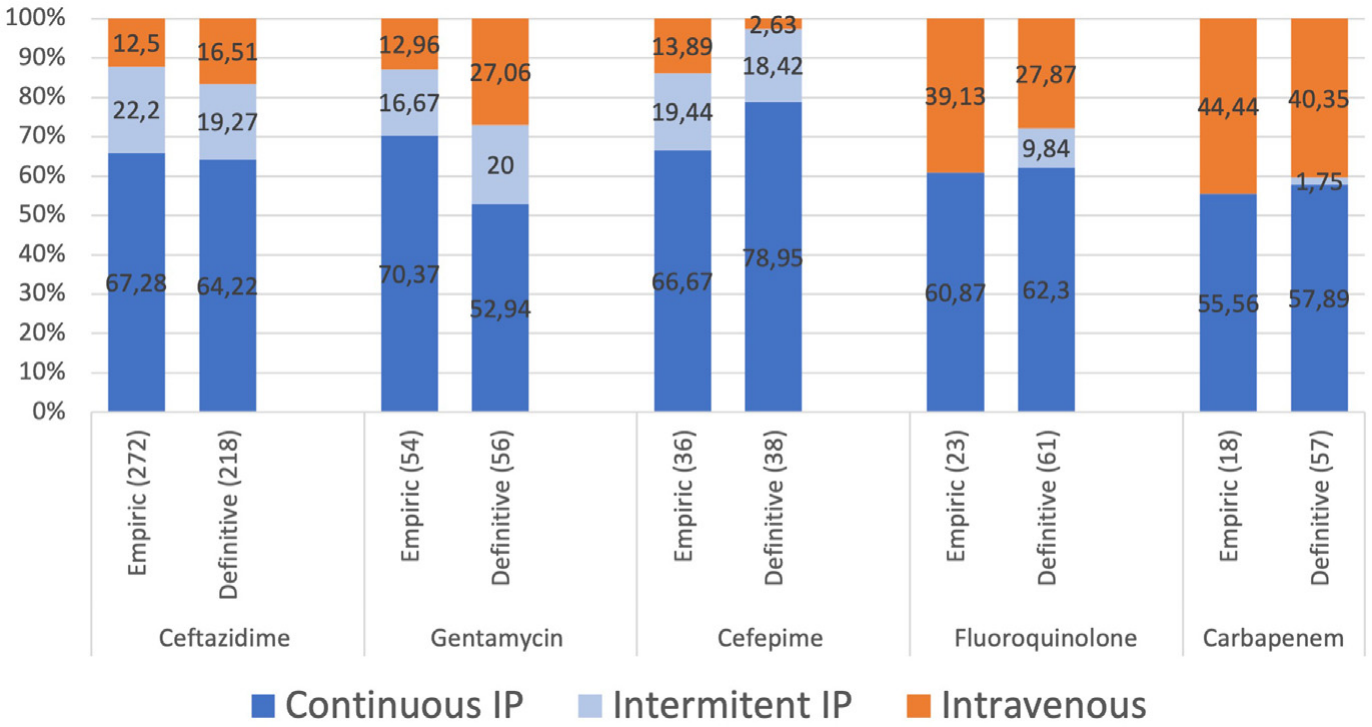
Route of administration of empiric and definitive treatment antibiotics for gram negative coverage∗. ∗Number of episodes treated given in brackets.

Clinical symptomatology by organism is presented in Figure 1a. The only discriminative parameter among Enterobacterales, *Pseudomonas* spp., *Acinetobacter* spp., and other gram-negative organisms was a higher disease severity score at presentation associated with *Pseudomonas* peritonitis than with other organisms (2.45 ± 1.1 vs. 2.13 ± 1.09, *P* = 0.04). Documented potential causes included preceding or concurrent exit site infection (40, 11%), poor hygiene (17, 4%) and accidental disconnection (16, 4%). In 259 cases (67%), no identifiable cause of GNP was reported. Empiric antibiotic therapy varied across centers, with 30 combinations reported (Table 2). The route of empiric antibiotic administration for gram-negative coverage is presented in Figure 2.

**Table 2:** Empiric therapy choice

Antibiotic	*n* (%)
Ceftazidime/vancomycin	121 (32%)
Ceftazidime/cefazolin	95 (25%)
Cefepime	38 (10%)
Gentamicin/vancomycin	22 (6%)
Fluoroquinolone/vancomycin	20 (6%)
Ceftazidime/gentamicin	18 (5%)
Ceftazidime	16 (4%)
Gentamicin/cefazolin	7 (1.5%)
Meropenem/vancomycin	7 (1.5%)
Meropenem	3 (0.5%)
Meropenem/fluoroquinolone	2 (0.5%)
Meropenem/gentamicin	2 (0.5%)
Cefuroxime/ceftazidime	2 (0.5%)
Cefuroxime	2 (0.5%)
Other or unspecified	24 (6.5%)

### Impact of Empiric Antibiotic Choice and Microbial Susceptibility on Primary Treatment Response

Data on early clinical response (abdominal pain and maximal temperature at 60–72 hours) were obtained for 269 peritonitis episodes (71%), whereas data on effluent cloudiness and cell count at 60 to 72 hours were obtained in 281 (74%) and 245 (65%) episodes, respectively (Figure1b). A good initial clinical response to empiric therapy was observed in 74% of these cases, with slightly lower rates for *Pseudomonas* spp. (36/56, 65%) than for other organisms (164/203, 77%; *P* = 0.06). Empiric ceftazidime was used exclusively for gram-negative coverage in 183 cases, cefepime in 31, and gentamicin in 19 cases. Initial responsiveness did not differ significantly for episodes in which gram-negative coverage included either cefepime (86%), gentamicin (73%), or ceftazidime (72%) (cefepime vs. ceftazidime or gentamicin; *P* = 0.09). Continuous i.p. administration of ceftazidime (*n* = 133) resulted in a slightly greater percentage of episodes exhibiting a good initial response as compared to intermittent i.p. ceftazidime (*n* = 38) administration (75% vs. 61%, *P* = 0.10).

Data on antibiotic susceptibility were available in 293 episodes (77%) overall and included cefazolin (*n* = 164), ceftazidime (*n* = 256), cefepime (*n* = 181) and aminoglycosides (*n* = 267) susceptibility (Table 3). For 199 episodes (52%), both antibiotic susceptibility and initial clinical response data were available.

**Table 3:** Antimicrobial susceptibility (n/number tested, %)

Type of organism	Cefazolin	Ceftazidime	Cefepime	Aminoglycoside
Enterobacterales	32/71 (45%)	73/97 (75%)	58/77 (75%)	96/110 (87%)
*Pseudomonas* spp.	16/36 (44%)	56/68 (82%)	39/43 (91%)	64/69 (93%)
*Acinetobacter* spp.	6/15 (40%)	34/43 (79%)	28/29 (97%)	44/47 (94%)
Other gram-negative	16/42 (38%)	37/48 (77%)	25/32 (78%)	34/41 (83%)
All organisms	70/164 (43%)	200/256 (78%)	150/181 (83%)	238/267 (89%)

A good initial clinical response was observed more frequently when the cultured bacteria were susceptible *in vitro* to any of the antibiotics given empirically (124/163, 76% vs. 21/36, 58%; *P* = 0.03). In a multivariate analysis accounting for age, dialysis duration, presence of ostomies, maximal body temperature at onset, severity of abdominal pain at presentation, *in vitro* bacterial susceptibility to empiric therapy and type of causative organism, only lack of severe abdominal pain at onset (OR: 3.39, 95% CI: 1.78–6.47; *P* = 0.0002) and *in vitro* bacterial susceptibility (OR: 3.36, 95% CI: 1.49–7.59, *P* = 0.004), were associated with a good initial treatment response.

### Definitive Therapy Choice, Treatment Duration, and Outcome

Ceftazidime (administered in 58% of cases alone or in combination), gentamicin (23%), fluoroquinolone (16%), carbapenems (15%), and cefepime (9%) were the most frequently prescribed antibiotics for definitive treatment after receipt of culture and susceptibility results. The route of antibiotic administration is presented in Figure 2. Monotherapy was prescribed in 181 cases (48%), most commonly with ceftazidime (*n* = 85, 47%), carbapenems (*n* = 21, 12%), cefepime (*n* = 16, 9%), fluoroquinolone (*n* = 15, 8%), gentamicin (*n =* 14, 8%), or cefazolin (*n* = 16, 9%).

The median duration of antibiotic therapy was 18 (IQR: 14–21, range: 8–42) days. Total therapy duration was shorter in patients who showed a good initial response to empiric treatment (18 vs. 20 days, *P* = 0.007). Evaluation of 246 non-*Pseudomonas* peritonitis episodes treated for 12 to 24 days showed FFR in 91% of 140 episodes treated for a median of 14 days (range 12–17), and in 88% of 106 episodes treated for a median 21 (range 19–24) days (*P* = 0.45). Multivariate analysis adjusting for age, initial clinical presentation, preceding exit site infection, and early treatment response confirmed the lack of impact of duration of antibiotic therapy on FFR (OR: 0.72, 95% CI: 0.30–1.73; *P* = 0.47). Catheter removal and temporary PD discontinuation were more common in episodes with preceding or coexisting exit site infection (OR: 3.79, 95% CI: 1.44–9.98, *P* = 0.007) and in patients aged <5 years at presentation (OR: 3.1, 95% CI: 1.24–7.74, *P* = 0.01).

Overall, 312 episodes (82%) were followed by FFR (including 23 with temporary PD discontinuation), whereas 27 (7%) continued PD with functional impairment (8 with temporary discontinuation). Forty patients (11%) terminated PD due to poor ultrafiltration capacity (*P* = 5), adhesions (*n* = 10), uncontrolled infections (*n* = 22), or secondary fungal peritonitis (*n* = 3). The FFR rate was 87% for continuous ambulatory PD and 90% for patients on automated PD (*P* = 0.8).

In multivariate logistic regression analysis, the odds of failure to achieve FFR and the odds of PD discontinuation increased with severe clinical symptomatology, *in vitro* resistance of the causative organism to empiric therapy and use of combination therapy was comparable to monotherapy in the definitive antibiotic scheme. In addition, *Pseudomonas* etiology was independently associated with failure to achieve FFR (Table 4).

**Table 4:** Multivariate risk factor analysis of failure to achieve full functional recovery, PD discontinuation and relapse

Variable	Lack of full functional recovery (with or without temporary PD discontinuation)		Permanent PD discontinuation		Relapse	
	OR (95% CI)	*P*	OR (95% CI)	*P*	OR (95% CI)	*P*
Peritonitis age below 5 yr	1.49 (0.70–3.13)	0.297	1.25 (0.48–3.25)	0.645	1.19 (0.42–3.38)	0.749
PD duration “ref < 12 mo”						
12–24 mo	1.1 (0.43–2.38)	0.270	0.64 (0.19–2.16)	0.071	1.06 (0.37–2.86)	0.163
>24 mo	2.40 (1.16–4.95)	0.008	3.08 (1.26–7.51)	0.001	1.26 (0.07–1.92)	0.134
Presence of any ostomy	1.25 (0.58–2.65)	0.568	1.45 (0.56–3.75)	0.438	0.61 (0.18–2.03)	0.339
Preceding or concurrent exit site infection	1.10 (0.43–2.79)	0.715	1.06 (0.35–3.38)	0.912	___	____
Severe abdominal pain at onset	3.81 (2.01–7.20)	<0.0001	3.56 (1.59–7.98)	0.002	2.51 (0.96–6.05)	0.058
Temperature >38.9 °C at onset	1.66 (0.84–3.26)	0.139	1.42 (0.62–3.25)	0.401	1.12 (0.35–3.55)	0.752
Severe abdominal pain at 60–72 h	3.94 (1.06–14.67)	0.040	3.56 (0.81–15.7)	0.093	___	____
Temperature >38.9 °C at 60–72 h	2.13 (0.36–12.38)	0.400	1.18 (0.16–8.60)	0.866	___	____
Etiology “ref = other GN”						
*Enterobacterales* spp.	0.78 (0.34–1.80)	0.824	0.74 (0.27–1.99)	0.902	0.76 (0.24–2.42)	0.893
*Pseudomonas* spp.	1.73 (1.71–4.21)	0.01	1.30 (0.43–3.65)	0.108	1.47 (0.39–5.61)	0.155
*Acinetobacter* spp.	0.44 (0.14–1.39)	0.052	0.27 (0.05–1.41)	0.097	0.36 (0.07–1.96)	0.148
*In vitro* bacterial resistance to empiric therapy	2.40 (1.21–4.79)	0.01	3.03 (1.34–6.86)	0.008	3.18 (1.15–8.79)	0.024
Monotherapy in postempiric scheme	0.40 (0.21–0.77)	0.006	0.24 (0.10–0.58)	0.002	1.13 (0.45–2.85)	0.788
Cumulative therapy duration 3 wk vs. 2 wk	1.58 (0.83–3.05)	0.164	1.54 (0.69–3.45)	0.287	1.59 (0.58–4.30)	0.363

CI, confidence interval; GN, gram-negative; OR, odds ratio; PD, peritoneal dialysis.

*P*-values <0.05 indicate statistically significant differences.

A total of 23 of 307 GNP episodes (7.5%) in which PD was continued were followed by a relapsing infection. In multivariate analysis, when holding other variables constant, susceptibility to empiric treatment decreased the risk of relapse by 72%. (Table 3).

An analysis of data pertaining to specific bacterial etiology was also conducted, with separate analyses for Enterobacterales, *Pseudomonas* spp., and *Acinetobacter* spp.

### Enterobacterales Peritonitis (*n =* 154)

There were 26 definitive treatment schemes reported. The most common were ceftazidime monotherapy (*n* = 38, 25%), ceftazidime/gentamicin (*n* = 14, 9%), ceftazidime/vancomycin (*n* = 11, 7%) and cefazolin monotherapy (*n* = 11, 7%). Median total therapy duration was 17 (IQR: 14–41) days. Overall, FFR was achieved in 127 Enterobacterales peritonitis episodes (82%). Comparison of 2-week (median: 14 days, range: 12–17) vs. 3-week (median: 21 days, range: 19–24) cumulative treatment duration with an effective antibiotic revealed no difference in the FFR rate (87% vs. 84%, *P* = 0.19). Nine cases of Enterobacterales peritonitis were followed by a relapse.

Monotherapy with a cephalosporin was prescribed in 56 maintenance treatment schemes in accordance with *in vitro* susceptibilities (38 ceftazidime, 7 cefepime, and 11 cefazolin). Median total treatment time was 17 (IQR: 14–17) days in the cefazolin group, 16 (IQR: 14–21) days in the ceftazidime group, and 16.5 (16.5–21) days in the cefepime group, respectively (*P* = 0.88). FFR was reported in 10 of 11 episodes (91%) treated with cefazolin (*8 Escherichia coli*, 3 *Klebsiella* spp.), as compared to 42 of 45 (93%) treated with ceftazidime or cefepime (14 *Klebsiella*, 13 *Enterobacter*, 11 *E coli*, 6 *Citrobacter*, 1 *Serratia*) (*P* = 0.78). There were 4 relapses in the patients treated with ceftazidime or cefepime, and none in the cefazolin treated patients (*P* = 0.29).

### *Pseudomonas* spp*.* Peritonitis (*n* = 82)

A total of 82 *Pseudomonas* spp. peritonitis episodes were evaluated. In half of these, 2 antipseudomonal antibiotics with different mechanisms of action were prescribed for maintenance antibiotic therapy. The combinations included ceftazidime/gentamicin (*n* = 18), ceftazidime/fluoroquinolone (*n* = 7), gentamicin/fluoroquinolone (*n* = 6), carbapenem/fluoroquinolone (*n* = 3), cefepime/gentamicin (*n* = 3), cefepime/fluoroquinolone (*n* = 2), and carbapenem/gentamicin (*n* = 2). In contrast, 29 episodes (37%) were treated with a single antipseudomonal agent, including ceftazidime (*n* = 15), gentamicin (*n* = 5), fluoroquinolone (*n* = 4), carbapenem (*n* = 3), and cefepime (*n* = 2). Five episodes were treated with a combination of 2 beta lactams, whereas another 7 episodes were treated with 1 or 2 agents that were not further specified. Those 12 episodes were excluded from further analyses. The final dataset consisted of 70 *Pseudomonas* spp. episodes, including 49 with initial clinical responsiveness data and 51 with information on effluent cloudiness at 60 to 72 hours. Twenty-nine episodes treated with monotherapy did not differ significantly from 41 episodes receiving dual antipseudomonal therapy with respect to initial clinical presentation (severe abdominal pain at onset: [12/29] 40% vs. [23/41] 57%, *P* = 0.14, and fever above 38.9 °C: [3/29] 10% vs. [16/41] 40%, *P* = 0.22), but more commonly demonstrated a good initial clinical response ([13/17] 76% vs. [20/32] 61%, *P* = 0.005) and absence of cloudy effluent at 60 to 72 hours of empiric therapy ([14/18] 78% vs. [15/33] 45%, *P* = 0.025). Median treatment duration was 15 (IQR: 14–21) days with monotherapy and 21 (IQR: 17–24) days in episodes treated with 2 antipseudomonal agents (*P* = 0.0007).

FFR was achieved in 23 of 29 episodes (79%) treated with 1 agent as compared to 22 of 41 (54%) episodes treated with combined therapy (*P* = 0.03). The fraction of episodes requiring catheter removal was slightly higher in those receiving 2 antipseudomonal agents (19% vs. 10%, *P* = 0.25). Multivariate analysis showed no difference in FFR associated with single versus dual therapy (OR: 0.31, 95% CI: 0.03–2.77, *P* = 0.29) after adjustment for age, clinical symptoms at onset, early treatment response, catheter removal, and treatment duration.

A comparison of 2-weeks versus 3-weeks treatment duration revealed that FFR was achieved in 61% of those treated for a median of 14 (range: 12–17) days compared to 75% in episodes treated for a median 21 (range: 19–24) days (*P* = 0.28).

### *Acinetobacter* spp. Peritonitis (*n* = 64)

Sixty-four *Acinetobacter* spp. peritonitis episodes were reported. Among 24 cases with further specification, 13 were *Acinetobacter baumannii*. Four episodes (6%) had a preceding or coexisting exit site infection. The symptomatology at onset and after 60 to 72 hours of empiric treatment is presented in Figure 1. Infections were most commonly treated with carbapenem, ceftazidime, and cefepime alone (55%) or in combination. Median cumulative treatment duration was 21 (range: 8–30) days. FFR was reported in 55 cases (85%), whereas 2 (3%) patients permanently discontinued PD.

## Discussion

Our registry review of 379 episodes of GNP in children was informative in terms of both management strategies and outcome data. We found substantial variation in empiric antibiotic regimens across dialysis programs with as many as 30 different combinations reported. *In vitro* bacterial susceptibility to empiric therapy was the only independent predictor of a good initial response. Although this is not surprising, it is exceedingly important because of increasing gram-negative antibiotic resistance. In our cohort, only 78% of the 256 infections in which ceftazidime susceptibility data was available, were susceptible to it. These data emphasize the importance of modifying empiric treatment regimens based on local susceptibility patterns, as well as patient-specific factors, which can be very challenging in regions with high rates of antibiotic resistance.

We found that *in vitro* susceptibilities were commonly reported for aminoglycosides, which is the class of antibiotics used in 13% of empiric treatment regimens. Importantly, recent United States Clinical Laboratory Standards Institute and European Committee on Antimicrobial Susceptibility Testing updates have resulted in significant changes in the interpretation of aminoglycoside susceptibility testing. Breakpoints for aminoglycosides for Enterobacterales have been lowered and gentamicin is no longer considered to be an effective therapy for *Pseudomonas* spp.^9^^,^^10^ By these newer standards, a larger proportion of isolates would likely be considered aminoglycoside resistant. In this context and considering the potential for aminoglycoside nephrotoxicity and the loss of residual kidney function in patients receiving PD, particularly in patients with mutations of the *MT-RNR1* gene, beta lactams seem to be a better option for empiric therapy.^11^ Cefepime, with an overall 83% *in vitro* susceptibility and with evidence of a good initial clinical response in 86% of treated episodes, may be a preferable empiric treatment choice compared to ceftazidime, though local treatment regimens should also consider availability, affordability, and local susceptibility patterns.

*Pseudomonas* spp. etiology, severe clinical presentation, *in vitro* resistance of the causative organism to empiric therapy, an inferior initial treatment response and dual- versus monotherapy as part of definitive management, increased the risk of failure to achieve FFR and PD discontinuation. Although severe presentation and antibiotic resistance to initial therapy as risk factors could have been predicted, the reasons why monotherapy in contrast to dual therapy was associated with a higher odds of FFR are less obvious. A possible explanation is that clinicians chose to use combined therapy in patients with more severe infections or with comorbidities, potentially affecting the outcome. In addition, and as previously described, PD duration more than 24 months, was an independent risk factor for treatment failure and lack to achieve FFR.^12^ Possible reasons might include an altered peritoneal immune defense function or increased peritoneal interstitial fibrosis affecting antibiotic absorption from the peritoneal cavity.^13^^,^^14^

Infections caused by *Pseudomonas* spp. also pose a therapeutic challenge due to the bacteria's ability to form a biofilm, reducing the chances of successful treatment without catheter removal.^15^ Often, peritonitis episodes are accompanied by a tunnel or exit site infection, further elevating the risk of subsequent technique failure.^16^^,^^17^ The 2012 pediatric and the current adult ISPD guidelines recommend combination therapy using 2 antibiotics with distinct mechanisms of action, both effective against *Pseudomonas* spp.^6^^,^^7^ This recommendation stems in part from the suboptimal outcomes observed in *Pseudomonas* peritonitis cases treated with a single agent reported in the Australia and New Zealand Dialysis and Transplant Registry (ANZDATA) database.^18^ However, it is important to note the limitations of this retrospective analysis, including the absence of adjustments for concomitant exit site or tunnel infections, disease severity, early response to antibiotic treatment, and treatment duration. In addition, the majority of patients were treated empirically with an aminoglycoside and later switched to ciprofloxacin upon isolation of *Pseudomonas aeruginosa* in culture. This may reflect the inferiority of aminoglycosides for the treatment of *Pseudomonas* rather than poor outcomes due to the number of agents used.

Contrary to the recommendation for dual therapy, our analysis showed no disparity in outcomes between 1-agent and 2-agent therapy of *Pseudomonas* spp. peritonitis, both in univariate and multivariate analysis, even after adjusting for age, disease severity score, 60 to 72-hour treatment response, and treatment duration. Furthermore, 75% of monotherapy patients in our study received beta-lactams. Our findings align with data from gram-negative infections in other body sites which indicate that not only does treatment with multiple antibiotics not result in better outcomes, but the practice is associated with increased toxicity; resultant guidelines in turn recommend use of a single agent, even in the case of *P aeruginosa* infections with difficult-to-treat resistance.^19^, ^20^, ^21^

Within our cohort, 91% of susceptible Enterobacterales isolates exhibited FFR when treated with cefazolin. This is comparable to the 93% recovery rate observed in those receiving ceftazidime or cefepime, with no reported relapses in the cefazolin group. Previously reported data from the International Pediatric Peritonitis Registry cohort had suggested a higher risk of relapse with cefazolin monotherapy compared to ceftazidime or aminoglycoside monotherapies.^22^ However, the International Pediatric Peritonitis Registry analysis did not specify the causative bacteria. A potential explanation for the previously observed inferior outcomes with cefazolin in treating *in vitro* susceptible organisms could be attributed to the historically recommended lower dose of cefazolin (15 mg/kg vs. 20 mg/kg recommended since 2012), now recognized as inadequate.^23^ Our analysis, accounting for higher cefazolin doses, does not support the association between cefazolin and inferior outcomes. In addition, there are many advantages to choosing cefazolin for susceptible infections; it is well-tolerated and cost-effective, and, owing to its narrow spectrum of activity, it is less likely to induce antimicrobial resistance than third-generation cephalosporins.^24^

The published adult and pediatric ISPD guidelines currently recommend a 3-week duration of antibiotic therapy for all gram-negative organisms^6^^,^^7^; however, there are no studies showing better outcomes with 3 weeks versus 2 weeks of therapy. In fact, we also found that there was no difference in FFR from peritonitis in cases of non-*Pseudomonas* GNP treated with 2 weeks versus 3 weeks of antibiotics. Our data, along with the risks associated with prolonged antibiotic therapy such as development of antimicrobial resistance, adverse events, and cost of therapy, favor the use of a 2-week duration for non-*Pseudomonas* GNP. Clinicians may consider longer durations of therapy if patients are slow to improve or there is concern for a concomitant exit site or tunnel infection, though these factors may also lead providers to consider catheter removal for source control. The use of shorter durations of treatment is also consistent with emerging evidence supporting shorter durations of therapy for gram-negative infections in general, including serious infections such as bacteremia.^24^ In contrast, in *Pseudomonas* spp. peritonitis, there was a trend toward improved FFR associated with more prolonged therapy, achieved in 75% of patients treated for a median 21 days and 61% for those treated for a median of 14 days. Although not statistically significant, this trend suggests a preferred 3-week treatment duration for *Pseudomonas* spp. peritonitis, independent of disease severity.

This study has limitations due to its observational nature precluding causal inference. Empiric and subsequent therapy scheme and length may have been modified depending on clinical presentation and comorbid conditions that have not been captured. Some potential risk factors for unfavorable outcome, such as presence or lack of antifungal prophylaxis, were not collected and could not be incorporated in the analysis. Similarly, the registry does not collect data on carbapenem susceptibility. The strength of the study is related to the collection of treatment and outcome data on a substantial number of GNP episodes in children, along with global nature of the data.

In conclusion, severe clinical manifestations, poor response to empiric treatment and resistance to empiric therapy were associated with failure to achieve FFR in pediatric patients with GNP. *Pseudomonas* peritonitis was accompanied by more severe symptoms at presentation compared to non-*Pseudomonas* infections, with an increased likelihood of failure to achieve FFR. Nevertheless, using 2 antipseudomonal agents did not increase the odds of a good outcome. For peritonitis caused by Enterobacterales that were susceptible to cefazolin, treatment with cefazolin resulted in similar outcomes as treatment with broader spectrum antibiotics. In response to the global crisis of rising antibiotic resistance, our data provide evidence that treatment of GNP with narrower spectrum antibiotics and shorter durations of therapy is not associated with inferior treatment outcomes, provided that treatment is in accordance with susceptibility data. This information will serve as an important contribution to the updated ISPD pediatric guidelines for the prevention and treatment of peritonitis in children.

## Appendix

The following Principal Investigators are active contributors to the International Pediatric Peritoneal Dialysis Network Registry: **Argentina:** L. Alconcher, Hospital Interzonal General, Bahia Blanca, P.A. Coccia, Hospital Italiano de Buenos Aires, A. Suarez, Hospital de Nińos Sor. Maria Ludovica La Plata, Patricia G. Valles, Hospital Pediatric Humberto Notti, Mendoza; **Canada:** Ch. Licht, Hospital for Sick Children, Toronto; **Chile:** F. Cano, Hospital Luis Calvo Mackenna, Santiago, M.A. Contreras, Roberto del Rio Hospital, Santiago. China: H. Xu, Children's Hospital of Fudan University, Shanghai. **Colombia:** J.J. Vanegas, Instituto del Rinon, Medellin; L.M. Higuita, Baxter Servicio al Cliente Colombia, Medellin.**China:** Yihui Zhai, Children’s hospital of Fundan University, Shanghai; E. Chan, Princess Margaret Hospital, Hong Kong; **Czech Republic**: K. Vondrak, University Hospital Motol, Prague. **Finland**: J. Lauronen, Hospital for Children and Adolescents, Helsinki**. France**: B. Ranchin, Hôpital Femme Mčre Enfant, Lyon; A.Zaloszyc, Children's Dialysis Center, Strasbourg; Ch. Samaille, Hospital Jeanne de Frandre, Lille; M. Fila, Pediatric Nephrology Unit, Montpellier, I. Vrillon, CHRU, Nancy, S. Tellier,Dialyse Pediatricue CHU, Toulouse. **Germany**: J.Thumfart, Charité Virchow-Klinikum, Berlin; L.Weber University Hospital, Cologne, Cologne; R. Büscher, Children's Hospital Essen; CP. Schmitt, F. Schaefer, D.Borzych-Duzalka Center for Pediatrics and Adolescent Medicine, Heidelberg; G. Klaus, KfH Kidney Center, Marburg;. **Greece:** V. Askiti, A&P Kyriakou Children’s Hospital, Athens, F. Papacristou, Aristoteles University, Thesaloniki. **Hungary:** AJ. Szabo, Semmelweis University, Budapest**. India:** N. Kamath, St. John’s Medical College, Bangalore, B. Basu, NRS Medical College & Hospital, Kolkata; A. Bagga, All India Institute of Medical Sciences, New Delhi**, Iran:** N. Hooman, Iran University of Medical Sciences, Tehran. **Italy:** F. Paglialonga, S. Testa, Fondazione Ospedale Maggiore Policlinico, Milano; E. Verrina, G. Gaslini Institute, Genova; S E. Vidal, Pediatric Nephrology, Dialysis and Transplant Unit, Padova; G. Leozappa, Department of Nefrologia-Urologia, Roma, **Republic of Korea:** Hee Gyung Kang, Seoul National University Children's Hospital, Seoul; **The Netherlands**: JW Groothoff, Academic Medcial Center, Amsterdam, **Malaysia:** YN Lim, Kuala Lumpur Hospital, Kuala Lumpur. **Macedonia**: E. Sahpazova Pediatric Clinic, Skopje **Nicaragua**: Y. Silva, Hospital Infantil de Nicaragua, Managua. **Oman:** M. Al Ryami, Royal Hospital, Muscat. **New Zealand:** R. Erickson, Starship Children's Hospital, Auckland. **Peru**: R. Loza Munarriz, Cayetano Heredia Hospital, Lima. Poland: A.M. Zurowska, D. Borzych-Duzalka, Medical University, Gdansk; D. Drozdz, Jagiellonian University Medical College; M. Szczepanska, Dialysis Division for Children, Zabrze, Ł.Obrycki, Children’s Memorial Health Institute, Warsaw; **Philippines:** A. Marbella, National Kidney and Transplant Institute, Quezon City. **Portugal**: T. Francisco, Hospital D. Estefania, Lisboa. **Saudi Arabia**: J. Kari, King Abdul Aziz University Hospital, Jeddah. **Serbia:** D. Kruscic, University Children’s Hospital, Belgrade. **Singapore:** H.K. Yap, Shaw-NKF-NUH Children's Kidney Center. **Spain:** G. Ariceta, University Hospital Materno-Infantil Vall d'Hebron, Barcelona. **Sweden:** L. Swartz, Barnkliniken, Lund. **Turkey:** A. Duzova, Hacettepe University, Ankara; S. Bakkaloglu, Gazi University, Ankara; I. Bilge, Department of Pediatric Nephrology, Çapa-Istanbul; Onder Yavascan Tepecik Children and Research Hospital, Izmir; S. Mir, Ege University Faculty of Medicine, Izmir-Bornova. **United Arab Emirates:** E. Simkova, Al Jalila Hospital, Dubai. **United Kingdom**: R. Shroff, Great Ormond Street Hospital, London; C. Ried, Evelina Hospital, London. A. Kaur, Royal Manchester Hospital, Manchaster; **Uruguay:** J. Grünberg, SE.N.NI.AD, Montevideo. **United States:** H. Patel, Nationwide Children's Hospital, Columbus; B.A. Warady, Children's Mercy Hospital, Kansas City; M. Lee, The University of California, San Francisco; M. Rheault, University of Minnesota, Amplatz Children's Hospital, Minneapolis; M. Pradhan, The Children's Hospital of Philadelphia, Philadelphia; J. Flynn, Seattle Children's Hospital, Seattle; C. Wong, Lucile Packard Children’s Hospital, Palo Alto.

## Disclosure

All the authors declared no competing interests.

## Supplementary Material

Supplementary File (PDF) **STROBE Checklist. STROBE STATEMENT.** The study followed the Strengthening the Reporting of Observational Studies in Epidemiology (STROBE) reporting guidelines.
